# Supplementary material for: “Outcomes of COVID-19 infection in patients with hematological malignancies- A multicenter analysis from Pakistan”
Source: PLoS One. 2022 Apr 21;17(4):e0267139. doi: 10.1371/journal.pone.0267139 (PMC9022796; doi:10.1371/journal.pone.0267139)
Supplement: Attachment — Submitted filename: PONE-D-21-33433_reviewer.pdf [file pone.0267139.s002.pdf]

PLOS ONE

Outcomes of COVID-19 infection in patients with hematological malignancies- A multicenter analysis from Pakistan

--Manuscript Draft--

**Manuscript Number:**	PONE-D-21-33433
**Article Type:**	Research Article
**Full Title:**	Outcomes of COVID-19 infection in patients with hematological malignancies- A multicenter analysis from Pakistan
**Short Title:**	Outcomes of COVID-19 infection in patients with hematological malignancies
**Corresponding Author:**	Adeeba Zaki, MBBS, FCPS<br>Aga Khan University Hospital<br>Karachi, PAKISTAN
**Keywords:**	Hematological malignancy; Outcome; Mortality; Hospital
**Abstract:**	Purpose<br><br>COVID-19 infection resulting from (SARS-CoV-2), began to spread across the globe in early 2020. Patients with hematologic malignancies are supposed to have increased risk of mortality from COVID-19 infection. From Pakistan, we report the analysis of the outcome and interaction between patient demographics and tumor subtype and COVID -19 infection and hematological malignancy.<br><br>Patients and Methods<br><br>This is a multicenter, retrospective study included adult patients with history of hematological malignancy who were tested positive for COVID-19 presented at the oncology department of 5 tertiary care hospitals in Pakistan from February 2020 to August 2020. The primary objective was to determine overall clinical outcome, the patient characteristics, clinical presentations, treatments administered, and mortality rate stratified by age, type of malignancy and oncological treatment status for COVID-19 in patients with hematological malignancy.<br><br>Results<br><br>107 patients with hematological malignancy and COVID -19 positive presented to the hospital during study period. The median age was 35 years. The most represented malignancies were acute leukemia (28.9%), non-Hodgkin's lymphomas (28.9%) with predominantly B cell lymphomas and Hodgkin's lymphoma. Most frequently symptoms were respiratory (41%), fever (32.7%) and diarrhea (4.6%). Around 45.8% patients were admitted to the hospital for acute care while 54.2% had mild disease and were advised home isolation. Overall mortality of the entire cohort was 28%, of which 51% were admitted in a hospital. When stratified for age, increased mortality was reported with age greater than 50 years (10.2%) and those with acute leukemia (9.3%). In addition, a mortality rate of 19.6% was seen in those who were on active oncological treatment.<br><br>Conclusion Taken together, this data supports the emerging consensus that patients with hematologic malignancies experience significant morbidity and mortality resulting from COVID-19 infection.
**Order of Authors:**	Adeeba Zaki, MBBS, FCPS
	Danish Hassan Khan
	Hassan Shaharyar
	Raheel Iftikhar
	Ayaz Mir

Powered by Editorial Manager® and ProduXion Manager® from Aries Systems Corporation

	Zeba Aziz
	Khatija bano
	Hafsa Naseer
	Qammarunisa Chaudhry
	Syed Waqas Imam Bokahri
	Salman Muhammad Soomar Ranani
	Munira Shabbir Moosajee
**Additional Information:**	
**Question**	**Response**
**Financial Disclosure**<br><br>Enter a financial disclosure statement that describes the sources of funding for the work included in this submission. Review the submission guidelines for detailed requirements. View published research articles from *PLOS ONE* for specific examples.<br><br>This statement is required for submission and **will appear in the published article** if the submission is accepted. Please make sure it is accurate.<br><br>**Unfunded studies**<br>Enter: *The author(s) received no specific funding for this work.*<br><br>**Funded studies**<br>Enter a statement with the following details:<br>• Initials of the authors who received each award<br>• Grant numbers awarded to each author<br>• The full name of each funder<br>• URL of each funder website<br>• Did the sponsors or funders play any role in the study design, data collection and analysis, decision to publish, or preparation of the manuscript?<br>• **NO** - Include this sentence at the end of your statement: *The funders had no role in study design, data collection and analysis, decision to publish, or preparation of the manuscript.*<br>• **YES** - Specify the role(s) played.<br><br>* typeset	No Funding received
**Competing Interests**	NO authors have competing interests

Use the instructions below to enter a competing interest statement for this submission. On behalf of all authors, disclose any competing interests that could be perceived to bias this work—acknowledging all financial support and any other relevant financial or non-financial competing interests.<br><br>This statement is required for submission and **will appear in the published article** if the submission is accepted. Please make sure it is accurate and that any funding sources listed in your Funding Information later in the submission form are also declared in your Financial Disclosure statement.<br><br>View published research articles from *PLOS ONE* for specific examples.<br><br>**NO authors have competing interests**<br><br>Enter: *The authors have declared that no competing interests exist.*<br><br>**Authors with competing interests**<br><br>Enter competing interest details beginning with this statement:<br><br>*I have read the journal's policy and the authors of this manuscript have the following competing interests: [insert competing interests here]*<br><br>* typeset	
**Ethics Statement**<br><br>Enter an ethics statement for this submission. This statement is required if the study involved:<br><br>• Human participants<br>• Human specimens or tissue<br>• Vertebrate animals or cephalopods<br>• Vertebrate embryos or tissues<br>• Field research	Ethical approval was obtained from Ethical Review Committee of the Aga Khan University Hospital, Karachi, Pakistan

Powered by Editorial Manager® and ProduXion Manager® from Aries Systems Corporation

Write "N/A" if the submission does not require an ethics statement.

General guidance is provided below. Consult the submission guidelines for detailed instructions. **Make sure that all information entered here is included in the Methods section of the manuscript.**

Format for specific study types

Human Subject Research (involving human participants and/or tissue)

- Give the name of the institutional review board or ethics committee that approved the study
- Include the approval number and/or a statement indicating approval of this research
- Indicate the form of consent obtained (written/oral) or the reason that consent was not obtained (e.g. the data were analyzed anonymously)

Animal Research (involving vertebrate animals, embryos or tissues)

- Provide the name of the Institutional Animal Care and Use Committee (IACUC) or other relevant ethics board that reviewed the study protocol, and indicate whether they approved this research or granted a formal waiver of ethical approval
- Include an approval number if one was obtained
- If the study involved *non-human primates*, add *additional details* about animal welfare and steps taken to ameliorate suffering
- If anesthesia, euthanasia, or any kind of animal sacrifice is part of the study, include briefly which substances and/or methods were applied

Field Research

Include the following details if this study involves the collection of plant, animal, or other materials from a natural setting:

- Field permit number
- Name of the institution or relevant body that granted permission

Powered by Editorial Manager® and ProduXion Manager® from Aries Systems Corporation

**Data Availability**<br><br>Authors are required to make all data underlying the findings described fully available, without restriction, and from the time of publication. PLOS allows rare exceptions to address legal and ethical concerns. See the PLOS Data Policy and FAQ for detailed information.<br><br>A Data Availability Statement describing where the data can be found is required at submission. Your answers to this question constitute the Data Availability Statement and **will be published in the article**, if accepted.<br><br>**Important:** Stating 'data available on request from the author' is not sufficient. If your data are only available upon request, select 'No' for the first question and explain your exceptional situation in the text box.<br><br>Do the authors confirm that all data underlying the findings described in their manuscript are fully available without restriction?	Yes - all data are fully available without restriction
**Describe where the data may be found in full sentences. If you are copying our sample text, replace any instances of XXX with the appropriate details.**<br><br>• If the data are **held or will be held in a public repository**, include URLs, accession numbers or DOIs. If this information will only be available after acceptance, indicate this by ticking the box below. For example: *All XXX files are available from the XXX database (accession number(s) XXX, XXX.*).<br>• If the data are all contained **within the manuscript and/or Supporting Information files**, enter the following: *All relevant data are within the manuscript and its Supporting Information files.*<br>• If neither of these applies but you are able to provide **details of access elsewhere**, with or without limitations, please do so. For example:	The data is available to the corresponding authors and can be provided on the reasonable request.

Data cannot be shared publicly because of [XXX]. Data are available from the XXX Institutional Data Access / Ethics Committee (contact via XXX) for researchers who meet the criteria for access to confidential data.<br><br>*The data underlying the results presented in the study are available from (include the name of the third party and contact information or URL).*<br>• This text is appropriate if the data are owned by a third party and authors do not have permission to share the data.<br><br>* typeset	
Additional data availability information:	

Manuscript
Click here to access/download;Manuscript;Multicenter cancer COVID19.doc

Title:

"Outcomes of COVID- 19 infection in patients with hematological malignancies- A multicenter analysis from Pakistan"

Adeeba Zaki[1], Danish Hasan Khan[2], Hasan Shaharyar[3], Raheel Iftikhar [4], Ayaz Mir [5], Zeba Aziz [6], Khatija Bano [5], Hafsa Naseer [6], Qamar un Nisa Chaudhry[4], Syed Waqas Imam Bokhari[3], Salman Muhammad Soomar[1], Munira Shabbir-Moosajee [1].

[1]Aga Khan University Hospital, Karachi Pakistan

[2]Hangzhou Tigermed Pakistan

[3]Shaukat Khanum Memorial Cancer Hospital and Research Centre, Lahore, Pakistan

[4]Armed Forces Bone Marrow Transplant Centre Rawalpindi, Pakistan

[5]Shifa International Hospital Pakistan

[6]Hameed Latif Hospital, Lahore, Pakistan

Author's Affiliation:

1) Dr. Adeeba Zaki, Senior Instructor, Department of Oncology, The Aga Khan University Hospital, Karachi, Pakistan.
2) Danish Hassan Khan, Clinical Project Manager (CPM Pakistan), Hangzhou Tigermed Pakistan
3) Dr. Hassan Shaharyar, Consultant Medical Oncologist, Shaukat Khanum Memorial Cancer Hospital and Research Centre, Lahore, Pakistan.

4) Dr. Raheel Iftikhar Consultant, Armed Forces Bone Marrow Transplant Centre Rawalpindi, Pakistan.

5) Dr. Ayaz Mir Director, Bone Marrow & Stem Cell Transplant, Shifa International Hospital Karachi, Pakistan

6) Dr. Zeba Aziz, Medical Oncologist, Hammed Latif Hospital, Lahore, Pakistan

7) Dr. Khatija Bano, Fellow clinical Hematology Shifa International Hospital Islamabad. Pakistan.

8) Dr. Hafsa Naseer, Postgraduate Resident, Hameed Latif Hospital Lahore, Pakistan

9) Dr. Qamarun nisa Chaudhry, Consultant, Armed Forces Bone Marrow Transplant Centre Rawalpindi, Pakistan

10) Dr. Syed Waqas Imam Bokhari, Medical Oncologist, Shaukat Khanum Memorial Cancer Hospital and Research Centre, Lahore, Pakistan

11) Salman Muhammad Soomar Research Specialist Department of Oncology, The Aga Khan University Hospital, Karachi, Pakistan.

12) Dr. Munira Shabbir Moosajee, Associate Professor, Department of Oncology, The Aga Khan University Hospital, Karachi, Pakistan

Corresponding Author:

Dr. Adeeba Zaki, Senior Instructor Department of Oncology. The Aga Khan University Hospital, National Stadium Road, Karachi 74800, Pakistan. Email: adeeba.zaki@aku.edu

ABSTRACT

Purpose

COVID-19 infection resulting from (SARS-CoV-2), began to spread across the globe in early 2020. Patients with hematologic malignancies are supposed to have increased risk of mortality from COVID-19 infection. From Pakistan, we report the analysis of the outcome and interaction between patient demographics and tumor subtype and COVID -19 infection and hematological malignancy.

Patients and Methods

This is a multicenter, retrospective study which included adult patients with a history of hematological malignancy who were tested positive for COVID-19 presenting at the oncology department of 5 tertiary care hospitals in Pakistan from February 2020 to August 2020. The primary objective was to determine overall clinical outcome, the patient characteristics, clinical presentations, treatments administered, and mortality rate stratified by age, type of malignancy and oncological treatment status for COVID-19 in patients with hematological malignancy.

Results

107 patients with hematological malignancy and COVID -19 positive presented to the hospital during study period. The median age was 35 years. The most represented malignancies were acute leukemia (28.9%), non-Hodgkin's lymphomas (28.9%) with predominantly B cell lymphomas and Hodgkin's lymphoma. Most frequently symptoms were respiratory (41%), fever (32.7%) and diarrhea (4.6%). Around 45.8% patients were admitted to the hospital for acute care while 54.2% had mild disease and were advised home isolation. Overall mortality of the entire cohort was 28%. Of which 51% were admitted in hospital. When stratified for age, increased mortality was reported with age greater than 50 years (10.2%) and those with acute leukemia

(9.3%). In addition, a mortality rate of 19.6% was seen in those who were on active oncological treatment.

Conclusion:

Taken together, this data supports the emerging consensus that patients with hematologic malignancies experience significant morbidity and mortality resulting from COVID-19 infection.

Keywords: Hematological malignancy, Outcome, Mortality, Hospital.

INTRODUCTION

The SARS-Cov-2 or COVID-19 also known as novel coronavirus has become a global threat and healthcare concern. Since its outbreak in China at the end of 2019, the pandemic has affected more than a 100 million people worldwide.[1] Although the outbreak is likely to have started from a zoonotic spread, it soon became clear that person-to-person transmission occurs mainly through respiratory droplets and direct contact with diseased person or indirect contact with fomites in the enviroment.[2-3] Many people have mild symptoms while others have no symptoms at all, but still actively carry and transmit the virus. However, some do develop severe symptoms such as respiratory failure, cytokine release syndrome, and multi-organ failure[4].

Since COVID-19 began to spread across the globe in early 2020, patients with co-morbidities and cancer are more susceptible to marked complications of viral infection. Cancer patients are more prone to increased risk of infections than individuals without cancer because of immunosuppression by the malignancy itself and anticancer treatments, such as chemotherapy or surgery.[5-6] and have a poorer prognosis. Cai et al have reported that patients with metastatic disease, hematological malignancy or lung cancer are at particularly high risk of severe

complications, requiring intensive care unit (ICU) admission, invasive mechanical ventilation and even death. Furthermore, active treatment such as surgery and immunotherapy are associated with a significantly increased risk (hazard ratios of 6.22 and 4.82) respectively, for poor outcomes,[7] Zhang et al reported a higher rate of adverse events (53.6 %) and mortality (28.6 %) in those who had their last anti-tumor treatment within 14 days of the infection (HR = 4.079, 95 % CI 1.086–15.322, P = 0.037). We do know that patients with hematological malignancies can have an underlying immune dysfunction and are vulnerable to viral and other infections.[9]Additionally, treatments which include cytotoxic agents, immunomodulators, hematopoietic stem cell transplantation, and chimeric antigen receptor T-cell therapy, are profoundly immunosuppressive. In addition, patients with hematologic malignancies have multiple risk factors of particular concern in the context of COVID-19 infection such as advanced age, underlying or treatment-induced comorbid illnesses like hypertension and diabetes, and chronic lymphopenia. These factors make this patient population particularly susceptible to an adverse outcome.

Public health measures have been universally instituted to control the disease spread and aim to decrease preventable hospital admissions. In addition it is recommended that patients receiving anticancer treatment should have vigorous screening for COVID-19.[8] However, cancer care encompasses a diverse array of primary tumor types and stages, affecting all age groups of patients, with different prognosis and outcomes. Therefore, labelling all patients with cancer as susceptible to COVID-19 is probably neither reasonable nor informative.

For patients with hematologic malignancies, overall risk of morbidity and mortality resulting from COVID-19 infection, as well as how this risk varies as a function of age, disease status,

type of malignancy, and cancer therapy is being studied. However, data from low- and middle-income countries is sparse. It is hypothesized that lower rates of testing and contact tracing and dearth of medical facilities adequately equipped to manage complicated covid infections can potentially result in higher morbidity and mortality in this high-risk population.

From Pakistan, we report the analysis of the interaction between patient demographics and tumor subtype and COVID -19 infection and outcomes in patients with hematological malignancy. This is a multicenter analysis from five tertiary care hospitals in Pakistan, all of whom have an established cancer center.

METHODS

We retrospectively collected data on all the patients with a history of hematological malignancies that tested positive for COVID-19 by RT-PCR and presented at the oncology department of 5 tertiary care hospitals in Pakistan: Aga Khan University Hospital Karachi, Armed Forces Bone Marrow Transplant Centre Rawalpindi, Hameed Latif Hospital Lahore, Shifa International Hospital Islamabad and Shaukat Khanum Memorial Cancer Hospital Lahore from February 2020 to August 2020. Demographic, clinical, treatment and laboratory data and serial samples for viral RNA detection were extracted from medical records.

Patients with a clinical or radiological diagnosis of COVID-19, without a positive RT-PCR test were not included in this analysis.

Outcomes

The primary objective of the study is to determine overall clinical outcomes of COVID-19 infection in patients with hematological malignancy. Secondary objectives of the study are to

determine the patient characteristics, clinical presentations, treatments administered, and mortality rate stratified by age, type of hematological malignancy and oncological treatment status.

Statistical Analysis

The data was entered and analyzed by using SPSS version 23. Calculated medians for all continuous variables and frequencies with percentages for categorical variables. Chi-square test was performed to check the association between the age and mortality.

RESULTS

Baseline characteristics

From February till August 2020, we identified 107 patients with hematological malignancy who presented to the hospital with a positive COVID-19 PCR test. Most of the patients' entries were from Shaukat Khanum Memorial Cancer Hospital (50%) followed by Shifa International Hospital (20%), Aga Khan University Hospital (16%), Armed Forces Bone Marrow Transplant Centre Rawalpindi (11%), Hameed Latif Hospital Lahore (3%). 49 (45.8%) patients were admitted to the hospital for acute care while 54.2% had mild disease and were advised home isolation.

Baseline patient's characteristics are shown in (Table 1). At the time of COVID-19 diagnosis, median age was 35 years (14-68). 39% of patients were younger than 30 years, while 34% and 28% were between age 30- 50 years and age >50 years, respectively. Two-thirds of the patients were female (67%). About 79% of patients had no comorbidities

other than the hematological malignancy. Other co-morbidities included hypertension in 17%

and diabetes in 9% of the patients. The most common malignancies were acute leukemia (28.9%), Non-Hodgkin's lymphoma (28.9%) with predominantly B cell lymphomas and Classical Hodgkin's lymphoma. Most of the patients were receiving active treatment (63%), mainly chemotherapy (41.1%), chemo-immunotherapy (16.8%), tyrosine kinase inhibitors (4.7%), and radiation therapy (0.9%). 8 patients had a prior history of stem cell transplant of which 7 were on immunosuppressive drugs. The median time to a positive covid PCR for patients on active treatment was 19 days (range 3-43 days).

Table1: Baseline characteristics of the study population.

**Demographic Characteristics of Patients**		
**Age (years) Range**	35 (14 – 68)	
**Sex**	n	%
Male	35	(32.7%)
Female	72	(67.3%)
**Co-morbidity**		
Diabetes	9	(8.4%)
Hypertension	17	(15.8%)
Ischemic Heart disease	1	(0.9%)
Chronic Kidney disease	1	(0.9%)
**No - comorbid**	79	(73.8%)
**Primary Malignancy**		
**ALL**	14	(13.1%)

**AML**	13	(12.2%)
**CML**	5	(4.6%)
**CLL**	7	(6.5%)
**SLL**	1	(0.9%)
**HCL**	1	(0.9%)
**Hodgkin's lymphoma**	19	(17.7%)
**Non-Hodgkin's lymphoma**		
B cell lymphoma	25	(23.3%)
T cell lymphoma	4	(3.73%)
**MDS**	4	(2.8%)
**Multiple myeloma**	8	(7.4%)
**HLH**	1	(0.9%)
**ITP**	1	(0.9%)
**Histiocytic sarcoma**	1	(0.9%)
**On Active cancer treatment**		
Yes	68	(63.6%)
No	39	(36.4%)
**Chemo immunotherapy**	**18**	**(16.8%)**
R CHOP	9	(50%)
R- Bendamustine	2	(11%)
R-ICE	3	(16.6%)

R-CEOP	1	(5.5%)
R-EPOCH	2	(11%)
Rituximab	1	(5.5%)
**TKI**	**5**	**(4.7%)**
**IV chemotherapy**	**44**	**(41.1%)**
CVP	1	(2.2%)
Idarubicin + cytarabine	10	(22.7%)
BFM protocol	4	(9.09%)
HyperCVAD	9	(20.4%)
ABVD	10	(22.7%)
Bendamustine	2	(4.54%)
VRD	4	(9.09%)
Cyclophoshamide +bortezomib	2	(4.54%)
Lenalidomide + carfilzomib	2	(4.54%)
**Radiation**	**1**	**(0.9%)**
**Symptoms at onset**		
Fever	35	(32.7%)
Respiratory symptoms	44	(41.1%)
GI symptoms	5	(4.6%)
Others	5	(4.6%)

Asymptomatic	18	(16.8%)

Presenting symptoms and treatment of COVID-19

As shown in Table 1, we investigated presenting features of all patients with COVID-19 infection. The most common presenting symptoms were respiratory (41% - cough, dyspnea), fever (32.7%) and diarrhea (4.6%), while 16.8% patients were asymptomatic. In terms of exposure, about 69.2% of patients did not report to have any known contact or travel exposure history; however, 16% and 14% reported to have travel and contact exposure, respectively. 75% of the 49 patients admitted to the hospital were classified as moderate to severe covid infection. 80% of these patients fulfilled the criteria of cytokine release syndrome. Lymphopenia was seen in 40% of these patients. The most common COVID19–specific therapies in our dataset were symptomatic treatment with steroids (60%) and anticoagulation (35%). Tocilizumab was used in 6.5%, remdesivir in 2.8% and hydroxychloroquine 3.7% of patients. Of the 49 hospitalized patients, 9% and 15% of the patients needed noninvasive and invasive ventilation respectively.

Outcomes of COVID-19 infection

Overall mortality of the study cohort was 28%. Of the 107 patients, 49 patients (45%) required admission to the hospital. The mortality rate in the admitted patients was 51%. 17 patients were transferred to intensive care unit and the mortality rate amongst these patients was 86%. 46.9% recovered from their illness and were discharged home. Average length of hospital stay was 12 days (1-38 days). When stratified for age, the mortality was 7.4%, 5.6% and 10.2% in the age groups 10-30 years, 31-50 years, and 51-70 years respectively. In our cohort the highest

mortality was seen in patients with acute leukemia (12.1%) followed by Non-Hodgkin's lymphoma (5.6%), chronic leukemia (3.7%) and multiple myeloma (3.7%). In addition, a mortality rate of 19.6% was seen in those who were on active oncological treatment. (Table 2) Mortality rate in patients receiving intravenous chemotherapy alone was about 14% and 4.6% receiving chemoimmunotherapy. Subsequent PCR data in the infected patients was available for only 17 patients. In those, the average time to a negative PCR result was 19 days (6-40 days).

Table 2: Stratification of Mortality

**Overall Mortality**		
	N	**DEATH**
Overall patients	107 (100%)	30 (28%)
Hospital admission	49 (45.8%)	25 (51%)
Home isolation	58 (54.2%)	0 (0.0%)
**Mortality stratified by Age**		
10 -30 yrs	41 (38.3%)	8 (7.4%)
31-50 yrs.	37 (34.5%)	6 (5.6%)
51-70 yrs.	29 (27.1%)	11(10.2%)

**Mortality stratified by type of malignancy**

**Disease**	Total	Death
**Acute Leukemia**<br>ALL<br>AML/MDS	31<br>(28.9%)<br>14<br>(17.7%)<br>17<br>(11.2%)	13 (12.1%)<br>4 (3.2%)<br>9 (8.5%)
**Chronic Leukemia**<br>CLL<br>CML	13 (12%)<br>8 (7%)<br>4 (3.7%)	4 (3.7%)<br>3 (2.8%)<br>1 (0.9%)
**HCL**	1(0.9%)	1 (0.9%)
**Hodgkin's lymphoma**	19<br>(17.7%)	3 (1.8%)
**Non-Hodgkin's lymphoma**	31 (27%)	6 (5.6%)
**Multiple Myeloma**	7 (7.4%)	4 (3.7%)
**HLH**	1 (0.9%)	0 (0.0%)
**ITP**	1 (0.9%)	0 (0.0%)
**Mortality stratified by active oncological**		

**treatment**		
Yes	68 (63%)	21 (19.6%)
**Mortality stratified by type of treatment**		
TKI	5 (4.7%)	1 (0.9%)
Intravenous chemotherapy	44 (41.4%)	15 (14%)
Chemo immunotherapy	18(16.8%)	5 (4.6%)
Radiation	1 (0.9%)	0 (0.0%)
Surveillance	39 (36.4%)	4 (3.7%)

DISCUSSION

Worldwide, health-care systems are facing an uphill task of dealing with the COVID-19 pandemic, a situation that is going to remain a challenge to all clinicians. The incidence of covid-19 and outcomes in cancer patients is a topic of great interest. It is evident now that the covid-19 will be a global health care issue for the foreseeable future, and it is imperative that clinicians understand the complexity of presentations and outcomes of patients with concomitant health issues that make them vulnerable for severe complications. Our study has mainly focused on outcomes of COVID-19 with hematological malignancies in a resource constrained environment and it’s the first multicenter analysis from Pakistan.

ASH Research Collaborative COVID-19 Registry analysis states that patients with hematologic malignancies have a higher mortality resulting from COVID-19 than in the general population. They reported an overall mortality of 28%, increasing to 42% in patients with moderate to severe infection.[10-11-12] Recently published study from Italy stated a mortality of 37% patients with hematological malignancy and COVID-19, with higher risk amongst those with older age, progressive disease, or severe infection [13-14]. Another multicenter study, analyzing outcomes in cancer patients from China reported mortality of 20%, with 41% mortality in the 22 patients with

hematologic malignancies.[15] UK Myeloma Forum published their results of 75 patients with COVID-19 and multiple myeloma, with a mortality of 55%.[16-17] In our study, the overall mortality mirrors the data the of these studies with an overall mortality of 23% and increasing to approximately 51% in the hospitalized patients and 86% in ICU patients. 50% of deaths were seen in patients younger than 50 years. This is most likely due to the fact that approximately 70% of our patients were in that age group. Therefore, this is likely over represented. It was interesting to note the relatively low numbers of patients of older age admitted to the hospital in our cohort. It could be hypothesized that treatment was deferred for the older patients with multiple co-morbidities in the initial months of the pandemic if they had a relatively stable clinical course. However, it would be interesting to study the outcomes of patients who had their treatment deferred.

Several other findings from our cohort are noteworthy. We analyzed the demographics of COVID-19 patients with hematological malignancy and explored the effect of cytotoxic chemotherapy and various chemo immunotherapy and targeted treatments on the trajectory of COVID-19. The incidence of COVID-19 was found to be more frequent in acute leukemias (29%) followed by non-Hodgkin's lymphoma (27%) and Hodgkin's lymphoma (18%)[18]. Furthermore, an increase in mortality has been reported in myeloid malignancies (MDS/AML/MPN) than lymphoid neoplasms (NHL/CLL/ALL/MM/HL) (43% vs. 35%), [19-20] which is similar to that seen in our study population. Majority of our study patients were on active treatment and reported a mortality rate of 19.6% in contrast to 10% among patients on surveillance. Interestingly a higher mortality rate (14%) was seen in patients receiving chemotherapy alone compared to a 4.6% receiving chemoimmunotherapy.

The most common COVID-19–specific therapies in our dataset were symptomatic treatment with steroids and anticoagulation, tocilizumab (6.5%), remdesivir (2.8%), hydroxychloroquine (3.7%). The use of tocilizumab and remdesivir in our cohort were low when compared to other studies.[10] One reason is that our data collection started in the early days of the pandemic when these drugs were not used regularly. Additionally, the availability of the drugs was sparse until the mid of 2020. Remdisivir was given emergency use authorization in May 2020 and gained full FDA approval in October 2020. It only then has this drug become widely available for use.

Morbidity rates from COVID-19 in patients with cancer who are admitted to the hospital are high particularly in older patients and those with hematological malignancies. But not all cancer patients are affected equally. These findings allow clinicians to risk stratify their patients; whether symptomatic or not; and make decisions on social isolation and shielding at appropriate levels. Our data and many other studies demonstrate that patients with hematological malignancies, particularly acute leukemias, are at a high risk for severe COVID-19 infection and mortality. Therefore, preemptive testing, early recognition of infections and prompt management at a facility with expertise to manage complications is of paramount importance. In addition, protective measures such as universal masks, social distancing and shielding this susceptible population from COVID-19 exposure is mandatory. Many sites have implemented use of telemedicine to on-site physical distancing, and these practices should continue if COVID-19 prevalence remains high.

Our study has some limitations. Our analyses are based on patients with hematological malignancy who sought help from centers where they were receiving their treatment. Therefore, this cohort did not capture the outcomes of patients who presented for management at a different

hospital. This is particularly true for patients who live in another cities or towns and most likely obtained treatment closer to home. Also, we likely missed patients on long term follow-up who approached their local GP or hospital. We, too, were unable to capture those patients who were asymptomatic and found to have COVID-19 positive on screening. In addition, patients who are on hospice care were not reported or included in this study. Therefore, it is not possible to accurately quantify the burden of infection in patients with hematological malignancy. Nonetheless, this dataset provides the glimpse of outcomes of patients who presented to a tertiary care hospital in Pakistan where both state of art management for covid infection and the primary malignancy was available. Majority of our patients were on active treatment and these results help prognosticate; patients who require intensive care carry a very grim prognosis. Data such as ours are especially important in formulating guidelines that are country/region specific regarding management of covid infections in a specific subset of patients. This is of importance in guiding management decisions in situations where resources are limited, and medical care is not covered by private insurance.

The expertise in management of covid infection has evolved over the last year. Early use of dexamethasone, remdesivir and anticoagulation has resulted in improved outcomes. Nonetheless,

determine the incidence and severity of infections in cancer patients who have been adequately vaccinated.

In summary, this study of patients with hematological malignancy and COVID-19 accentuates several significant considerations for clinical care and emphasizes the urgent need for more data. Longer-term follow-up and larger sample sizes are needed to understand the effect of SARS-CoV-2 on outcomes in patients with hematological malignancy.

Abbreviations

CI- Confidence Interval

HR- Hazard Ratio

ICU- Intensive Care Unit

NHL- Non-Hodgkin's Lymphoma

RT-PCR- Real Time Polymerase Chain Reaction

UK- United Kingdom

Declaration

Competing interests:

No Potential conflict of interest exist.

Funding Disclosure

The authors have not received any kind of funding for this research study from any institution or agency.

Ethics approval:

Ethical approval was obtained from Ethical Review Committee of the Aga Khan University Hospital, Karachi, Pakistan.

Availability of Data and Materials

The data is available to the corresponding authors and can be provided on the reasonable request.

Author's contribution

A.Z.- Conceptualization, Writing original draft

D.K, S.S- Data Curation & Formal Analysis

H.S., R.I., A.M, Z.A. K.B- Methodology & Writing original draft

H.N., Q.C., S.B- Writing original draft

M.M- Supervision & Writing, reviewing final draft.

REFRENCES

1. Moujaess E, Kourie HR, Ghosn M. Cancer patients and research during COVID-19 pandemic: a systematic review of current evidence. Critical Reviews in Oncology/Hematology. 2020 Apr 22:102972.
2. Li Q, Guan X, Wu P, Wang X, Zhou L, Tong Y, Ren R, Leung KS, Lau EH, Wong JY, Xing X. Early transmission dynamics in Wuhan, China, of novel coronavirus–infected pneumonia. New England Journal of Medicine. 2020 Jan 29.
3. Ong SW, Tan YK, Chia PY, Lee TH, Ng OT, Wong MS, Marimuthu K. Air, surface environmental, and personal protective equipment contamination by severe acute respiratory syndrome coronavirus 2 (SARS-CoV-2) from a symptomatic patient. Jama. 2020 Apr 28;323(16):1610-2.
4. Waris A, Khan AU, Ali M, Ali A, Baset A. COVID-19 outbreak: current scenario of Pakistan. New Microbes and New Infections. 2020 Apr 14:100681.
5. Jiang AM, Shi X, Liu N, Gao H, Ren MD, Zheng XQ, Fu X, Liang X, Ruan ZP, Yao Y, Tian T. Nosocomial infections due to multidrug-resistant bacteria in cancer patients: a six-year retrospective study of an oncology Center in Western China. BMC Infectious Diseases. 2020 Dec;20(1):1-2.
6. Lièvre A, Bouché O, Aparicio T, Michel P, Pontchaillou CH, Reims CH, Rouen CH. Risque et gravité du COVID-19 chez les patients atteints de cancer digestif.
7. Cao Y, Li Q, Chen J, Guo X, Miao C, Yang H, Chen Z, Li C, Li L. Hospital emergency management plan during the COVID- 19 epidemic. Academic Emergency Medicine. 2020 Apr;27(4):309-11.

8. Oh WK. COVID-19 infection in cancer patients: early observations and unanswered questions. Annals of Oncology. 2020 Mar 31.
9. Fontana L, Strasfeld L. Respiratory virus infections of the stem cell transplant recipient and the hematologic malignancy patient. Infectious Disease Clinics. 2019 Jun 1;33(2):523-44.
10. Wood WA, Neuberg DS, Thompson JC, Tallman MS, Sekeres MA, Sehn LH, Anderson KC, Goldberg AD, Pennell NA, Niemeyer CM, Tucker E. Outcomes of patients with hematologic malignancies and COVID-19: a report from the ASH Research Collaborative Data Hub. Blood advances. 2020 Dec 8;4(23):5966-75.
11. ElGohary GM, Hashmi S, Styczynski J, Kharfan-Dabaja MA, Alblooshi RM, de la Cámara R, Mohmed S, Alshaibani A, Cesaro S, Abd El-Aziz N, Almaghrabi R. The risk and prognosis of COVID-19 infection in cancer patients: a systematic review and meta-analysis. Hematology/oncology and stem cell therapy. 2020 Jul 30.
12. Salunke AA, Nandy K, Pathak SK, Shah J, Kamani M, Kotakotta V, Thivari P, Pandey A, Patel K, Rathod P, Bhatt S. Impact of COVID-19 in cancer patients on severity of disease and fatal outcomes: a systematic review and meta-analysis. Diabetes & Metabolic Syndrome: Clinical Research & Reviews. 2020 Jul 28.
13. Mangone L, Gioia F, Mancuso P, Bisceglia I, Ottone M, Vicentini M, Pinto C, Rossi PG. COVID-19 patients and cancer in northern Italy. medRxiv. 2020 Jan 1.
14. Passamonti F, Cattaneo C, Arcaini L, Bruna R, Cavo M, Merli F, Angelucci E, Krampera M, Cairoli R, Della Porta MG, Fracchiolla N. Clinical characteristics and risk factors associated with COVID-19 severity in patients with haematological

malignancies in Italy: a retrospective, multicentre, cohort study. The Lancet Haematology. 2020 Oct 1;7(10):e737-45.

15. Yang K, Sheng Y, Huang C, Jin Y, Xiong N, Jiang K, Lu H, Liu J, Yang J, Dong Y, Pan D. Clinical characteristics, outcomes, and risk factors for mortality in patients with cancer and COVID-19 in Hubei, China: a multicentre, retrospective, cohort study. The Lancet Oncology. 2020 May 29.
16. Cook G, Ashcroft AJ, Pratt G, Popat R, Ramasamy K, Kaiser M, Jenner M, Henshaw S, Hall R, Sive J, Stern S. Real‐ world assessment of the clinical impact of symptomatic infection with severe acute respiratory syndrome coronavirus (COVID‐ 19 disease) in patients with Multiple Myeloma receiving systemic anti‐ cancer therapy. British Journal of Haematology. 2020 May 21.
17. Wang B, Van Oekelen O, Mouhieddine TH, Del Valle DM, Richter J, Cho HJ, Richard S, Chari A, Gnjatic S, Merad M, Jagannath S. A tertiary center experience of multiple myeloma patients with COVID-19: lessons learned and the path forward. Journal of hematology & oncology. 2020 Dec;13(1):1-2.
18. He W, Chen L, Chen L, Yuan G, Fang Y, Chen W, Wu D, Liang B, Lu X, Ma Y, Li L. COVID-19 in persons with haematological cancers. Leukemia. 2020 Jun;34(6):1637-45.
19. Yigenoglu TN, Ata N, Altuntas F, Bascı S, Dal MS, Korkmaz S, Namdaroglu S, Basturk A, Hacıbekiroglu T, Dogu MH, Berber İ. The outcome of COVID‐ 19 in patients with hematological malignancy. Journal of medical virology. 2020 Jan 1.
20. Mehta V, Goel S, Kabarriti R, Cole D, Goldfinger M, Acuna-Villaorduna A, Pradhan K, Thota R, Reissman S, Sparano JA, Gartrell BA. Case fatality rate of

cancer patients with COVID-19 in a New York hospital system. Cancer discovery. 2020 Jul 1;10(7):935-41.
