# Supplementary material for: “Outcomes of COVID-19 infection in patients with hematological malignancies- A multicenter analysis from Pakistan”
Source: PLoS One. 2022 Apr 21;17(4):e0267139. doi: 10.1371/journal.pone.0267139 (PMC9022796; doi:10.1371/journal.pone.0267139)
Supplement: Attachment — Submitted filename: Response to reviewer comments multicenter.docx [file pone.0267139.s004.docx]

Reviewer #1: The authors of the manuscript "Outcomes of COVID‐19 infection in patients with hematological malignancies- A multicenter analysis from Pakistan“ have partially addressed previous comments. The manuscript still needs intensive language editing.

Response: Grammatical corrections are made, see track changes

Reviewer #3: The authors have addressed most of the concerns from the 1st-round review, and this reviewer is satisfied with the improved quality of the revised manuscript. However, the English language skill still needs to be improved.

Response: Grammatical corrections are made, see track changes

Reviewer #4: Dear /sir, most of the comments given were addressed correctly. I have a few suggestions to improve the readability of this manuscript before publication is commenced. My concerns are:-
- Please expand the term COVID-19 and SARS-CoV-2 at the beginning of the abstract page 3 line 3

Response: Changed to Severe acute respiratory syndrome coronavirus 2 (SARS-CoV-2). Coronavirus disease of 2019 (COVID-19) page #3 line #3-4

- Replace …‘from February 2020 to August 2020’ with … from February to August 2020’ page 3 line 11.

Response: Changed to February to August 2020

- The information included in the operational definition must be cited with appropriate literature/s.

Response: Cited all operational definitions

- Authors should check for typos and grammatical errors throughout the manuscript which need to be edited after careful reading.

Response: Grammatical corrections are made, see track changes

Reviewer #5: I suggest the authors to keep the first 3 paragraph of Baseline characteristics, taking care to integrate the information added in the revised version to avoid repetitions.

Response: Made two sections added first 3 paragraphs with their tables in baseline characteristics and rest in multivariable analysis.

The comparative mortality and hospitalization statistics in the general population should be mentioned for the reader to make a fair comparison.

Response: However, in the general population it range from 9-29.6%. In white Hispanic group the morality was less than 10% while in Black and Hispanic groups it was less than 30% in the general population. Page 18 line# 6-8
